# Devenir objetos frente a la inteligencia artificial

**DOI:** 10.18294/sc.2025.5617

**Published:** 2025-07-30

**Authors:** Hugo Spinelli

**Affiliations:** 1 Doctor en Salud Colectiva. Investigador, Instituto de Salud Colectiva, Universidad Nacional de Lanús, Buenos Aires, Argentina. hugospinelli09@gmail.com Universidad Nacional de Lanús Instituto de Salud Colectiva Universidad Nacional de Lanús Buenos Aires Argentina hugospinelli09@gmail.com

**Keywords:** Inteligencia Artificial, Tecnocapitalismo, Deshumanización, Artificial Intelligence, Technocapitalism, Dehumanization

## Abstract

Este ensayo reflexiona críticamente sobre los efectos del avance de la inteligencia artificial (IA) en la subjetividad, el trabajo y las instituciones, retomando los aportes del pensamiento latinoamericano en ciencia y tecnología y las discusiones de la filosofía contemporánea. Entendemos que la IA representa un capítulo más, pero no menor, en el proceso histórico de deshumanización de la ciencia, al sustituir el pensamiento basado en lo relacional y lo simbólico por una lógica algorítmica que amenaza con convertir a los sujetos en objetos. Desde allí, se cuestiona el retorno de la idea de progreso, ahora sustentado en la IA, la cual, con su antihumanismo radical, pretende constituirse en una racionalidad hegemónica, anulando la acción política, la otredad y el lenguaje humano. Frente a este panorama, proponemos recuperar los modelos institucionales artesanales, pequeños y relacionales que resguarden lo humano frente al dominio tecnocrático, para ello recuperamos el pensamiento crítico de autores como Gadamer, Arendt y Sadin. El texto expresa un llamado urgente a reconfigurar nuestras prácticas sociales y políticas para enfrentar los efectos de la IA sobre la vida de los colectivos sociales.

## Abstract (en)

This essay offers a critical reflection on the effects of the advancement of artificial intelligence (AI) on subjectivity, labor, and institutions, drawing on contributions from Latin American thought on science and technology, as well as from contemporary philosophical debates. We understand AI as yet another - though not minor - chapter in the historical process of the dehumanization of science, as it replaces relational and symbolic thinking with an algorithmic logic that threatens to turn subjects into objects. From this perspective, the essay questions the resurgence of the idea of progress, now underpinned by AI, which - through its radical anti-humanism - seeks to establish itself as a hegemonic rationality, suppressing political action, otherness, and human language. In light of this scenario, we advocate for the recovery of small-scale, relational, and artisanal institutional models that safeguard the human against technocratic dominance. To this end, the text revisits the critical thought of authors such as Gadamer, Arendt, and Sadin. It constitutes an urgent call to reconfigure our social and political practices in order to confront the effects of AI on the life of social collectives.

## Introducción

### Humanismo, sociedad y tecnología

“Nuestra ignorancia está planificada por una gran sabiduría” Raúl Scalabrini Ortiz (1898-1959)

Este trabajo se interroga sobre nuestras condiciones de sujetos frente al devenir de la inteligencia artificial (IA). Y en el marco del simposio “*Teoria Crítica da Saúde Digital*”, nos preguntamos si este texto es una botella que arrojamos al mar desde la Isla de Itaparica frente al naufragio del humanismo, o es una invitación a la acción política^1^. 

Las relaciones entre sociedad, ciencia y tecnología fueron analizadas críticamente en América Latina entre las décadas de 1950 y de 1970, enmarcadas en lo que se conoció como el Pensamiento Latinoamericano de Ciencia y Tecnología (PLACTED), el Pensamiento Latinoamericano en Ciencia, Tecnología y Sociedad (PLACTS), o la Escuela Latinoamericana de Pensamiento Latinoamericano en Ciencia, Tecnología y Desarrollo (ELAPCyTED). Ese pensamiento surgió en diversos países, como respuesta a preocupaciones que vinculaban la ciencia y la tecnología con la sociedad. Estuvo integrado por científicos y pensadores que desde distintos encuadres institucionales, ideológicos y políticos trabajaron esa relación. El movimiento convocó a figuras tan prestigiosas como diferentes que provenían de la ciencia, el pensamiento cepalino o el marxismo. Entre sus integrantes se destacaron, en Argentina, Jorge Sábato, Amílcar Herrera, Oscar Varsavsky y Carlos Martínez Vidal; en Brasil, Helio Jaguaribe, Theotônio dos Santos, Fernando Enrique Cardoso, José Pelucio Ferreira y Fabio Erber; en México, Miguel Wionzcek y Víctor Urquidi; en Perú, Francisco Sagasti; en Chile, Osvaldo Sunkel; en Venezuela, Marcel Roche; en Uruguay, Máximo Halty Carrère, entre otros. La intención era poder enfrentar críticamente el desarrollo técnico-científico propuesto desde los países centrales. La agenda de entonces, aún vigente, proponía un nuevo orden tecnológico, que implicaba la autonomía tecnológica de los países, más allá de sus diferencias (Dagnino et al., 1996). Muy lejanos resultan esos propósitos ante la realidad marcada por el antihumanismo radical de la IA^2^.

## Ciencia y pensamiento

Los desarrollos científicos tienden a ser asociados a la idea del progreso que la modernidad ubica siempre en el futuro. Esa situación es repetitiva en la ciencia hegemónica, la cual subordina el pensamiento a las técnicas y a lo instrumental, que con sus destellos y brillos que encandilan, y producen “*parpadeos*” que impiden el pensar^3^^,^^4^. La ruptura epistemológica que plantea Heidegger con la invitación a pensar, la encontramos en el devenir del pensamiento de Mario Testa, quien abandona las ideas de la planificación en su libro *Pensar en salud*^5^. 

La lógica instrumental de la razón moderna^6^^,^^7^ oculta la pregunta que buscan responder las tecnologías y quienes las enuncian. ¿O acaso conocemos quiénes y cuáles fueron las preguntas que originaron la implementación de la IA?, ¿o estamos frente a un desarrollo tecnológico autónomo y meramente instrumental? La IA, con sus brillos, también produce parpadeos, al decir de Nietzsche, pero no tardaremos en descubrir que la IA no solo no cambia nuestras realidades sociales, sino que las empeoran y agravan^2^. La IA no tiene en cuenta los olvidos, ni las anulaciones epistémicas, en las que se basa, como tampoco las deudas sociales acumuladas, que se potenciarán con su implementación^2^.

La IA, en tanto expresión de la matriz racional de la modernidad^6^^,^^7^^,^^8^, se vuelve a presentar como “el uno que lo explica todo”, en post de un futuro de progreso, en el que no importan las consecuencias sociales de su implementación^2^. El objetivo de la IA es el de siempre: “el progreso”. Un progreso que nunca llega porque no solo no “derrama” a los sectores empobrecidos de nuestras sociedades, sino que los excluye. Las ideas del desarrollo solo han producido acumulación en muy pocos, y cada vez en menos sectores sociales. Los escenarios con la IA agravaran radicalmente las inequidades sociosanitarias

### ¿De qué hablamos cuando hablamos de inteligencia?

Los filósofos griegos no desarrollaron un concepto formal de inteligencia, pero sí tenían equivalentes lingüísticos que eran utilizados para caracterizar a una persona como inteligente, por ejemplo, el concepto de *synesis* (comprensibilidad), o el de *phronesis* (sabiduría práctica). Aristóteles aplicaba el concepto *phronesis* a los animales para describir, por ejemplo, el trabajo de las abejas y las hormigas, que demostraban un sentido del tiempo con sus actividades^9^. 

Gadamer, en su libro *El lado oculto de la salud*^9^, tiene un capítulo titulado “El problema de la inteligencia”, en el que se interroga sobre la naturaleza de dicho concepto y plantea que desde la filosofía se busca “conocer la experiencia previa del mundo que la inteligencia articula y encierra”^9^. Gadamer también se pregunta si es posible que la escasa antigüedad de nuestro concepto usual de inteligencia no sea más que un accidente del lenguaje^9^.

La ciencia que conocemos asocia inteligencia con la capacidad de explicar los fenómenos que ella estudia de manera clara y unívoca^9^. Gadamer ubica en la ilustración -siglos XVII a XVIII- el momento en que la inteligencia deja de ser la facultad de “reconocer los principios”, y pasa a centrarse y significar la capacidad de “reconocer los objetos”^9^. Para él, tanto en la vida cotidiana como en el lenguaje científico, las palabras nunca son autónomas, y reciben el significado de su cercanía con otras palabras. Así, en la vida cotidiana, se asocia inteligencia con agudeza, rapidez de captación y sagacidad. En cambio, la ciencia, a partir del siglo XVII, la entiende como la capacidad de reconocer objetos. Ante ello, Gadamer se pregunta si el concepto de inteligencia que utiliza la ciencia no encierra una decisión previa, que entiende se pueda referir como un prejuicio^9^. Gadamer no asocia la inteligencia con lo artificial, pero si la asocia con la *intelligentsia*^9^.

## La Intelligentsia y la inteligencia artificial

Asistimos a una realidad compuesta por viejos problemas sociales que se agravaron en el tiempo: el aumentos de la pobreza y la exclusión social, que expresan las grandes desigualdades sociales; la contaminación ambiental; las débiles democracias que se caracterizan como Estados sin ciudadanos^10^; sociedades estructuradas bajo lógicas patriarcales; y un dominio de la técnica sobre el pensamiento que busca acabar con él. Y esa es la tarea de la IA, ya que la *intelligentsia* considera como muy peligroso que las personas piensen, se pretende que los sujetos devengan objetos, cosas, autómatas.

### La intelligentsia

Se la concibe compuesta por intelectuales, científicos y grupos sociales cercanos a ellos, caracterizados por acumular mucho poder. La *intelligentsia*​ se involucra en complejas actividades mentales y creativas, orientadas al desarrollo y a la diseminación de la cultura dominante. El término *intelligentsia* denota una cierta categoría de intelectuales que son vistos y se presentan como custodios de la historia y la cultura. Para Gadamer, esa intelligentsia nos impone deberes, modas y convenciones sociales, y una cierta normatividad social^9^.

### La inteligencia artificial, o la versión digital de Funes el memorioso

La expresión IA fue utilizada por primera vez en 1955 por el matemático John McCarthy en New Hampshire, EEUU, al inaugurarse el movimiento de la cibernética, que se proponía crear una nueva disciplina destinada a simular y reproducir artificialmente algunos de los procesos del cerebro humano^2^.

El concepto de IA ya forma parte de discursos científicos y análisis económicos y políticos, pero sus riesgos éticos, morales y humanitarios permanecen invisibilizados por el parpadeo^2^^,^^3^. La IA se suma como un nuevo ejemplo de lo que Stephen Gould llamó *La falsa medida del hombre*, señalando las consecuencias de las interpretaciones basadas en el determinismo biológico sobre la inteligencia^11^.

La IA constituye una nueva apuesta para eliminar toda huella humana en la producción social^2^. Hay que desmitificar que la IA piensa. No, no piensa, solo relaciona algoritmos, lo cual nos permite afirmar que la IA es la versión digital de Funes el memorioso, título de un cuento de Borges que describe como Funes podía recordar todo, con los más mínimos detalles, pero no podía pensar^12^.

Gadamer manifiesta que no es su deseo negar la inteligencia como una facultad humana^9^, pero nos recuerda que la psicología ha discutido intensamente si existe una única capacidad humana que pueda llamarse inteligencia^13^. 

### De autómatas, robots y sociobiología

Consideramos a la IA como el último capítulo -por ahora- de un proceso histórico, científico, político e ideológico originado en la antigüedad, basado en la idea de los autómatas, a la que luego se sumaron otros capítulos como el hombre-máquina^14^, y la sociobiología^15^^,^^16^, por citar solo algunos de ellos.

Para Éric Sadin el hombre ha buscado siempre superar su “armadura biológica”, tratando de lograr la “reproducción antropomórfica” con poderes superiores^2^, y cita como ejemplo el Génesis donde se menciona que “Dios crea al hombre a su imagen y semejanza”^2^. Otro ejemplo está en el Talmud, con el Golem, el texto sagrado de la cultura judía que señala la imperfección de la creación del Golem, figura a la cual Jorge Luis Borges le dedica un poema^12^. También se encuentran autómatas en la mitología griega, por ejemplo, en la *Ilíada*, y en distintos momentos históricos en los que se buscó construir autómatas que ayudaran o sustituyeran a las personas en los procesos de trabajo. Las ideas de los *autómatas o robots* alcanzan su mayor desarrollo con el capitalismo. Los autómatas, en tanto juguetes, son representaciones del mundo con los cuales se juega en la infancia, pero en la vida adulta se transforman en una realidad opresiva^17^.

Veamos de manera resumida el desarrollo de la idea del hombre-máquina y de la sociobiología, y de la similitud de los objetivos que persiguieron con respecto a los de la IA. *L’homme machine*^14^ es el título del libro de Julien Offray de La Mettrie (1709-1751), médico y filósofo francés, escrito en la primera mitad del siglo XVIII. En ese texto, Offray asumía que los humanos eran animales complejos y que no tenían más alma que otros animales, y que los humanos operaban como máquinas, basándose para ello en las ideas de Descartes^18^.

La idea del hombre como máquina fue recuperado por la sociobiología, desde la genética, en las figuras de Edward Osborne Wilson^16^ y Richard Dawkins^15^. Así, la sociobiología se presentó como el estudio de las bases biológicas del comportamiento social, postulando la base genética de dicho comportamiento, siguiendo la hipótesis que la conducta de todo ser vivo y, por lo tanto, también del ser humano, está, en general, determinada genéticamente^19^. Wilson y Dawkins van a centrarse en la biología en desmedro de la cultura, tratando de subsumir la sociología dentro de la biología, lo cual tiene serias implicaciones políticas y culturales^19^.

Somos máquinas de supervivencia, vehículos autómatas programados a ciegas con el fin de preservar las egoístas moléculas conocidas bajo el nombre de genes. Esa es una realidad que me llena de asombro. A pesar de que lo sé desde hace años, me parece que nunca me podré acostumbrar totalmente a la idea. Una de mis esperanzas es lograr cierto éxito en provocar el mismo asombro en otros.^15^

La biología tiene una larga relación con el tema de la inteligencia, muchas de esas relaciones son analizadas críticamente por Stephen Gould^11^^,^^20^^,^^21^^,^^22^ y también por Achard^23^, quien en 1977 publica *Discours biologique et ordre social*, que es traducido al español y publicado en México en 1980, con prólogo de Hugo Mercer, en la colección Salud e Ideología que dirigía Eduardo Menéndez^23^. Al final del libro, bajo el nombre de “A título de Colofón”, Pierre Achard escribe:

Los capítulos anteriores resaltan el imperio de la referencia biológica en los más diversos dominios; medicina, sin duda, pero también psicología, economía, política nacional e internacional. En cada oportunidad esta referencia abre el camino para una generalización totalizadora de acuerdo con el discurso filosófico de los biólogos. El fenómeno tecnocrático que goza del prestigio general de la ciencia presenta, sin embargo, otro aspecto: en la línea de un pensamiento mecanicista promueve la fantasía de una administración máquina, justa en tanto que objetiva, imparcial en tanto que automática, “matemática” que suprimiría el problema político. Esta fantasía tiene su versión capitalista (programación lineal y búsqueda del único *optimum*) o socialista (remplazar por la administración de los bienes el gobierno de las personas). Estos dos fenómenos ideológicos tienen en común que dejan fuera de discusión el poder político: la referencia biológica naturaliza su existencia, el discurso tecnocrático naturaliza, por racionalización, sus decisiones.^23^

En 1995, Herbert Simon y Allen Newell Simon escribieron “Ahora hay máquinas que leen, aprenden y pueden crear”^24^, dando a entender que se había dado una solución al problema mente-cuerpo. Pero Searle en su texto *Mentes, cerebros y ciencia*^25^ atacó este pensamiento, con el experimento de la habitación china, en el que muestra cómo una máquina puede realizar una acción sin siquiera entender lo que hace y el porqué lo hace. Por lo tanto, para Searle la lógica usada por las computadoras no busca el contenido en la acción, que es la usada por los seres humanos y que da lugar a la construcción de sentidos^26^.

## Las epistemologías en juego

### La deshumanización de la ciencia en los campos sociales

Los grandes anuncios sobre la IA se inscriben en la discusión epistemológica de los siglos XVII y XIX -racionalismo e idealismo- representados por Descartes, Kant y Hegel, y las posiciones centradas en el ser, y el lenguaje desarrollado en las primeras décadas del siglo XX por Heidegger, que rompe con el esquema del sujeto que estudia un objeto. Esa discusión epistemológica se desarrolló en el marco de una progresiva deshumanización de la ciencia. Tomaremos tres pensadores alemanes para señalar esa deshumanización.

A principios del siglo XIX Guillermo Von Humboldt fundador y rector de la Universidad de Berlín^27^ señaló la pérdida del humanismo que representaban los progresos científicos, y postuló el desarrollo integral del individuo a través del humanismo y la investigación libre, criticando la educación que se enfocaba únicamente en la utilidad práctica, o en la mera acumulación de conocimientos sin considerar el desarrollo del carácter y la capacidad crítica del individuo^27^.

También en Alemania, pero un siglo más tarde, Heidegger reflexionó y estudio la existencia del ser y su relación con el lenguaje. La influencia de su pensamiento se expresó en sus discípulos y en quienes siguieron sus ideas: Hannah Arendt, Hans Gadamer, Maurice Merleau-Ponty, Paul Ricoeur, Emmanuel Lévinas, Jacques Derrida, Michel Foucault, Louis Althusser, Karl Jasper, Gilles Deleuze, Richard Rorty, Edith Stein, Jacques Lacan, Herbert Marcuse, Gianni Vattimo y Franco Basaglia entre otros. 

En la misma época y en el marco de una Alemania que se encaminaba hacia su destrucción, Walter Benjamín, señaló críticamente el proceso de mecanización centrado en la idea de que la máquina puede hacerlo todo, y como se ocultaba que esa dinámica provocaba atrofia, no solo muscular, sino también intelectual. Para Walter Benjamin, las máquinas empobrecían el comportamiento humano y así los seres humanos “viven su existencia como autómatas […] prefiguración de la inhumanidad futura”^28^. En síntesis, sostenemos que la ciencia cuando niega lo relacional y el lenguaje, cosifica al otro, lo cual explica la crisis del humanismo que caracteriza a la ciencia en su devenir histórico.

En la modernidad, el trabajo industrial y la fábrica se convirtieron en modelos universales para la sociedad, incluso para los campos sociales, desencadenando un proceso de deshumanización que domina en la mayoría de las instituciones sociales. 

### La negación de la ontología del trabajo en los campos sociales y el modelo institucional que deviene de esa negación

En su libro *Epistemologías del Sur*, Boaventura de Sousa Santos sostiene que debemos achicar el futuro y expandir el presente, que es el momento de la acción. Y, en ese presente, cumplir con las deudas del pasado, cuyo olvido no es accidental, sino que responde a los intereses del poder y el capital. 

Basados en esa premisa de Boaventura de Sousa Santos, vamos a recuperar, de manera sucinta, los señalamientos sobre la deshumanización de la ciencia, y discutir la ontología del trabajo en lo social, y sus modelos institucionales que no se corresponden con los modelos fabriles que dominan las áreas sociales. 

En la modernidad, la deshumanización de la ciencia se exacerbó en el marco de los paradigmas mecánicos, proceso en el cual el trabajo artesanal devino trabajo industrial, y el taller del artesano en fábrica^17^^,^^29^. El devenir de esos cambios, con sus graves consecuencias sociales, colocan en agenda la necesitad de otras concepciones del trabajo en lo social y nuevas institucionalidades para la acción territorial; acción que concebimos relacional y simbólica, basada en la premisa de Paulo Freire que “la cabeza piensa donde los pies pisan”^30^. Esa premisa invalida a la IA, que se propone acabar con todo proyecto que coloque a los sujetos en relación, en el marco de procesos intersubjetivos que son la esencia de lo social. La IA postula afirmaciones globales sin necesidad de contextualizar, eliminando lo cultural. Posición contraria a la expresaba por Oscar Varsavsky cuando sostenía la diferencia entre ciencia y cientificismo^31^.

En Brasil, algunas de las propuestas de humanización del cuidado en el campo de la salud realizadas por José Ricardo Ayres^32^, Lilia Blima Schraiber^33^, Suely Deslandes^34^, los desarrollos de Emerson Merhy de micropolíticas y trabajo vivo en acto^35^^,^^36^ y la búsqueda de formas de gestión humanizadas por parte de Gastão Campos^37^^,^^38^ son desarrollos que marcan una lógica contraria a lo instrumental que domina en el campo de la salud.

Entendemos a las instituciones sociales del Estado como una red social inclusiva, no tecnológica, sino humana, centrada en conversaciones sustentadas en el compromiso entre lo que se dice y lo que se hace, con la construcción de acuerdos para compartir horizontes^39^^,^^40^^,^^41^, con una fuerte impronta de lo simbólico, y con evidencias objetivas del impacto de las acciones en el territorio, para construir ciudadanía social^10^^,^^42^. 

La prioridad en las agendas sociales no debe ser la IA, sino la discusión sobre lo que entendemos por trabajar en las áreas sociales, y si debemos proponer modelos artesanales o modelos industriales, y cuáles debieran ser los modelos institucionales adecuados para esos trabajos. La omisión de esa discusión nos ha llevado a aspirar y naturalizar los modelos institucionalizados del mundo industrial, esa es la explicación de que tengamos grandes hospitales, grandes universidades, grandes tribunales, y grandes cárceles, en vez de llevarnos a luchar por pequeños centros de salud^43^, pequeñas universidades^44^^,^^45^, y pequeños juzgados y lugares de detención^46^^,^^47^^,^^48^ por citar solo algunos ejemplos. 

Esas “otras” propuestas debieran priorizar la praxis, el aprender sobre el enseñar, el cuidar sobre la atención, y la recuperación social sobre el encierro y el aislamiento social. Esas acciones deberían estructurarse en lógicas territoriales y no programáticas, en las que lo humano se ubique sobre lo técnico, y lo social sobre lo artificial^29^^,^^49^^,^^50^. 

Ernst Schumacher, intelectual y economista alemán, publicó en 1973 *Lo pequeño es hermoso: Economía como si la gente importara*^51^*.* Allí critica la orientación de la sociedad centrada en lógicas económicas, camino que también siguió la ciencia y la tecnología, en desmedro de las personas^51^. Schumacher postula otras institucionalidades a escala humana, y el que sean pequeñas no constituye un rasgo negativo, sino que las enaltece por su humanismo, sin afectar su calidad científica. 

Esas institucionalidades deberán reconocer y habilitar al *Homo ludens*^52^ como parte del trabajo y la vida institucional, ya que el juego es un elemento central en las micropolíticas que constituyen lo relacional. La hipótesis que sostenemos es que el tamaño de la organización es inversamente proporcional al desarrollo del juego en las instituciones^29^^,^^47^^,^^53^. El *Homo ludens* es negado en la relación *Homo sapiens*-*Homo faber*.

Postulamos otras institucionalidades, no porque sea fácil lograr consensos sobre esa propuesta, sino porque consideramos que es muy necesarias, lo cual no es sinónimo de fácil^54^. Las instituciones a escala humana favorecen los juegos, las prácticas y las micropolíticas, en cambio, si las instituciones crecen permanentemente comienzan a ser invadidas por procedimientos burocráticos, con la consiguiente despersonalización de los procesos que terminan por anular el juego y las personas. Frente a una realidad social que se ha ido complejizando, postulamos instituciones pequeñas en cuanto al tamaño, y al número de trabajadores, con una configuración rizomática que resguarde las micropolíticas que humanizan la vida institucional; aclarando que pequeñas no quiere decir pocas, sino muchas, múltiples, pero singulares y contextualizadas. 

Cuando el juego se vuelve serio, desaparece lo lúdico, y se fortalecen las dinámicas arbóreas con toques kafkianos que llevan a la desafiliación de lo público, no solo en los trabajadores, sino también en los colectivos del territorio que dejan de sentir a esas instituciones del Estado como propias, y empiezan a escuchar cantos de sirenas que les prometen lo que no les darán, pero que tampoco les dan las instituciones públicas. Vivimos momentos marcados por un fuerte desapego a lo público y a lo estatal, con una crisis de legitimidad de las instituciones públicas frente al avance de ideas no democráticas en gobiernos elegidos democráticamente en varios países de América Latina y de otros continentes. 

Las propuestas anteriores las ubicamos enfrentadas a la IA, que es presentada como una “pansolución” frente a las dificultades para dar respuestas adecuadas y de calidad a las demandas de las poblaciones, por ejemplo, en diferentes dimensiones del proceso salud-enfermedad-atención-cuidado (PSEAC):

El médico, el hospital, así como otras entidades cualificadas están condenadas a ser destronadas, incluso marginalizadas, por la emergencia de nuevos “jugadores” que pretenden erigirse como los interlocutores más aptos para garantizar nuestro seguimiento más perfecto, y alertarnos, casi en tiempo real, en relación con la inminencia de riesgos o patologías.^2^

## El antihumanismo radical de la IA

La incorporación de la IA al campo de la salud intenta anular toda racionalidad médica que no responda a la medicina científica occidental; incluso, dentro de esta última, reducir significativamente la atención por personas para pasar a ser atendidos por la IA, lo cual intenta medicalizar el 100% de los malestares y padecimientos. Mas allá de esta aberración, nos preguntamos, ¿cómo se financiará ese nuevo gasto?, ¿en cuantos puntos del PBI se incrementará el gasto en salud?; gasto que se caracteriza en todos los países por la ineficiencia e ineficacia y por lo complejidad que representa su control^55^.

En las primeras décadas del siglo pasado, Raúl Prébisch, un economista de ideas liberales y figura de la CEPAL, preocupado por el desarrollo de los países de América Latina se preguntaba ¿dónde va a trabajar la gente que el progreso técnico desaloje de la agricultura? Para él, la respuesta estaba en la industrialización de los países de América Latina para mejorar las relaciones de intercambio entre centro y periferia^56^^,^^57^. El aggiornamento de la pregunta de Prébisch es ¿de qué va a trabajar la gente que el “progreso” de la IA desaloje del mercado laboral?, ¿tenemos respuestas?, ¿es viable políticamente un *ingreso universal ciudadano* financiado por la rentabilidad de la IA?

En los últimos tiempos proliferan publicaciones y noticias sobre el “venturoso destino” que nos espera con la IA. También circulan y, cada vez más, publicaciones críticas de autores disimiles que avizoran tiempos muy oscuros^58^^,^^59^. 

Éric Sadin, en sus libros, notas y conferencias, lleva adelante una crítica muy fuerte contra la IA, señalando las graves consecuencias económicas, sociales y culturales que tiene y tendrá para la humanidad^2^, que constituyen, para él, una seria amenaza para el empleo de millones de trabajadores, ya que se estima que la IA podría reemplazar 2/3 del empleo a nivel mundial^2^^,^^60^^,^^61^. 

Para Sadin, lo digital dejó de ser una forma de almacenamiento, indexación y manipulación de datos, sonidos e imágenes, para transformarse en un enunciador de “la verdad”, la cual se estructura bajo algoritmos que le atribuyen a los procesadores cualidades humanas, que les permiten evaluar situaciones y sacar conclusiones^2^. Vivimos el ethos de una época generada por una arquitectura tecnológica, que no deja lugar a la palabra. ¿Humanidades digitales? se pregunta Sadin^2^. El proyecto de la IA pretende duplicar la estructura del cerebro de manera artificial, y para ello utiliza conceptos como: chips “sinápticos”, “neuromórficos”, “redes de neuronas artificiales”, “procesadores neuronales” que se propagan por las mal llamadas redes sociales, que para Sadin nada tienen de sociales, y por ello propone llamarlas “plataformas experienciales de uno mismo”^2^. La IA constituye una racionalidad alimentada por enunciados preformateados que empobrecen el lenguaje, y pretenden la automatización de la acción humana, la cual quedaría reducida a esquemas y ecuaciones, que niegan la producción simbólica. Así la IA pasaría a constituir una racionalidad con la capacidad de formular verdades^2^. Todo ello lleva y constituye una crisis de la cultura.

Sadin entiende que entramos a la era antropomórfica de la técnica, la cual toma un camino que él divide en tres tipos: aumentado, parcelario y emprendedor. El primero busca modelarse sobre nuestras capacidades cognitivas, pero presentándolas como palancas a fin de elaborar mecanismos que, inspirados en nuestros esquemas cerebrales, están destinados a ser más rápidos, eficaces, y fiables que los que nos constituyen naturalmente. El segundo, antropomorfismo parcelario, está destinado a garantizar tareas específicas, y por último el antropomorfismo emprendedor, capaz de acciones automatizadas en función de conclusiones delimitadas. Este triple devenir antropomórfico de la técnica pretende, a largo plazo, una gestión sin errores de la cuasi totalidad de los sectores de la sociedad^2^.

Sadin se interroga sobre cómo pensar nuestras acciones frente a la IA en relación con los derechos sociales, con su regulación, con la justicia social, con la soberanía política, con la independencia económica y las cuestiones éticas. Todos temas que requieren de un Estado fuerte y comprometido con los derechos sociales, si se pretende continuar un proyecto civilizatorio, al decir de Sergio Arouca en relación con la Reforma sanitaria en Brasil; proyecto que no desplace a la mayoría de las poblaciones a situaciones de miseria y abandono. Pero esos temas no constituyen problemas para los agentes de la IA. ¿Se resolverán?, ¿cuáles serán las consecuencias de su falta de resolución?

Es indudable que la IA ofrece un proceso de trabajo más barato, rápido y flexible desde una mirada economicista. Pero ello no implica un proyecto de sociedad, excepto que el proyecto de sociedad acepte la idea de que 2/3 de las personas vivan sin ingresos fijos, o con un ingreso mínimo universal que naturalice la exclusión social. Parece que para los que impulsan la IA, la gran mayoría de los habitantes del mundo, están de más^2^.

Frente a la IA nos despertamos tarde, nos adormecidos por los encandilamientos tecnológicos de las últimas décadas. Y cuando nos estamos despertando, nos damos cuenta de que hemos sido olvidados y solo nos queda aspirar a convertirnos a un individualismo tirano replegado sobre uno mismo, de manera que mientras la vida transcurre, nos transformemos en espectadores sentados, en el mejor de los casos, en un sillón o, lo más probable, en una caja.

Mientras vivimos en el mundo de la “posverdad”, “de las *fake news*” y de la idea de la revolución cultural^62^, escenarios propios del anarcoliberalismo, la IA pretende anular nuestras capacidades de pensar, de renunciar al propio lenguaje que se transformara en un pseudo lenguaje, en el marco de una relación ciega con el presente. Vamos hacia marcos impuestos a través de textos sin sentimientos, con una desvalorización de la palabra. Textos que Sadin adjetiva como “amputados”, “necrosados”, “clonados”, “esquematizados”, y “sintetizados”^2^. ¿Vamos a aceptar esas consecuencias falsamente civilizatorias en detrimento de nuestras capacidades generativas?

Vivimos tiempos en los que prevalece el rencor en un mundo sin fe y sin ley, en los que la IA con su lógica algorítmica vulnera pactos internacionales a los cuales han adherido la mayoría de los países, como los derechos sociales^2^. ¿Es esta realidad el logro del proyecto civilizatorio de la modernidad?^2^. Sadin recupera el planteo de Pier Paolo Pasolini, quien sostenía la importancia de “decir que no”^2^. Sadin nos invita a no resignarnos, a ser actuantes, a decir ¡esto no lo queremos!, ¡esto si los queremos!, a no renunciar a la subjetividad, ni a la pluralidad, ni a la otredad. A dejar testimonios de lo que está pasando, testimonios que buscarán evitar que la IA se naturalice a la par de que no abandonemos la idea de un mundo mejor, más justo, menos desigual y para todos^2^.

### Ideas para el sentirjugarhacerpensar

Vamos a cerrar este texto recuperando el pensamiento de Hannah Arendt^63^^,^^64^, a quién Sadin cita frecuentemente^2^. Esa valiente y brillante mujer, convocó en sus escritos al accionar humano, al cual consideró el único hacedor del destino, a la vez que se oponía a la verdad sistemática, a la que consideró despótica, y responsable de la crisis de la cultura; verdad, representada en este texto, por la IA. Esto nos lleva a preguntarnos sobre el proyecto de sociedad que propone la IA, ya que no habrá complementariedad entre ella y los trabajadores, entre ella y las personas. Es tan urgente como necesario dar testimonio, de esta realidad que nos proponen, y convertirla en acción política^2^. 

Ante los escenarios tan obscuros, como terribles que nos plantea la IA, vamos a recurrir a la mitología para recordar que, en el fondo de la caja de Pandora, los dioses habían dejado a*Elpis,* -el espíritu de la esperanza-, de donde proviene el dicho popular “la esperanza es lo último que se pierde”. Y a esa esperanza la depositamos en la acción política^1^^,^^63^^,^^64^*.*

Aceptamos a la IA para los procesos mecánicos y repetitivos que lleven a la alienación a los trabajadores, tanto en el campo industrial, como en los campos sociales. Esos trabajadores desplazados deberán tener como mínimo asegurado un ingreso ciudadano que garantice una vida digna. Pero rechazamos el ingreso de la IA en los campos sociales, que buscan anular los procesos relacionales y las identidades culturales en post de una homogeneidad de la sociedad que anula las múltiples expresiones de lo subjetivo y de lo cultural. 

La discusión sobre la IA no debe, ni puede, omitir preguntarse sobre el lugar que ocupan en su implementación, la ética, la otredad, lo relacional y lo cultural. Omitir esas relaciones tendrá consecuencias sociales muy graves, profundizando las desigualdades sociales, y la deshumanización de la ciencia.

La IA representa una seria amenaza para las relaciones entre sociedad, ciencia y tecnología que, como señalamos se buscaron desarrollar en América Latina ente las décadas de 1950-1970. Debemos recuperar y volver a pensar esos textos. Y si el problema es la deshumanización, debemos dejar de pensar el trabajo sobre la base de modelos industriales, para pensarlo sobre la base de modelos artesanales^17^^,^^29^, donde la idea de taller sustituya la idea de fábrica. 

Por todo ello, convocamos a un cambio radical de los modelos institucionales en todas las áreas sociales, para lo cual proponemos la construcción de una nueva hegemonía institucional basada en instituciones pequeñas y múltiples en una estructura de autonomías dentro de autonomías que abandonen el modelo fabril y que en el territorio se centren en lo relacional, la manera humana de enfrentar a la IA.
